# Electroacupuncture at Zusanli and at Neiguan characterized point specificity in the brain by metabolomic analysis

**DOI:** 10.1038/s41598-020-67766-0

**Published:** 2020-07-01

**Authors:** Der-Yen Lee, Yu-Rung Jiu, Ching-Liang Hsieh

**Affiliations:** 1 Graduate Institute of Integrated Medicine, College of Chinese Medicine, China Medical University, Taichung, 40402 Taiwan; 2 School of Chinese Medicine, College of Chinese Medicine, China Medical University, Taichung, 40402 Taiwan; 3 Chinese Medicine Research Center, China Medical University, Taichung, 40402 Taiwan; 4 Graduate Institute of Acupuncture Science, College of Chinese Medicine, China Medical University, 91 Hsueh-Shih Road, Taichung, 40402 Taiwan; 5 Department of Chinese Medicine, China Medical University Hospital, Taichung, 40447 Taiwan

**Keywords:** Biological techniques, Metabolomics

## Abstract

Different point stimulations can induce brain activity in specific regions, and however whether these stimulations affect unique neurotransmitter transmission remains unknown. Therefore, we aimed to investigate the effect of point specificity to the brain by resolving the metabolite profiles. Eighteen Sprague–Dawley rats were randomly divided into three groups: (1) the sham group: sham acupuncture at Zusanli (ST36) without electric stimulation; (2) the Zusanli (ST36) group: electroacupuncture (EA) at ST36; and (3) the Neiguan (PC6) group: EA at PC6. Then, the metabolites from rat brain samples were measured by LC–ESI–MS. The results of a partial least squares discriminant analysis revealed the differences among the sham, ST36, and PC6 groups regarding the relative content of metabolites in the cerebral cortex, hippocampus, and hypothalamus. EA at PC6 resulted in downregulation of adenosine, adrenaline, γ-aminobutyric acid, glycine, and glutamate majorly in hippocampus, and then in cerebral cortex. Otherwise, EA at ST6 resulted in upregulation of adrenaline and arginine in hippocampus, and all stimulations showed barely change of identified neurotransmitters in hypothalamus. These differential metabolite and neurotransmitter profiles prove that brain areas can be modulated by point specificity and may provide a maneuver to understand more details of meridian.

## Introduction

Acupuncture stimulation can affect brain activity, including the somatosensory system, motor area, and limbic system; additionally, it can modulate the activity of specific brain areas. Hence, different points produce different effects, which is a phenomenon referred to as point specificity^1^. Different points on the same meridian produce brain activity in similar area. For instance, acupuncture at the liver meridian Taichong (LR3) and Zhongdu (LR6) induces activity in the ipsilateral superior parietal lobe^2^. Acupuncture at adjacent points LR3 and Neiting (ST44) at different meridians induces distinct cerebral activation patterns, and this accounts for the mechanism of point specific therapeutic effects^3^. Overall, acupoints are specific to induce certain downstream modulation.

A possible mechanism of acupuncture is the regulation of dopamine release to affect other neurotransmitters such as γ-aminobutyric acid (GABA), glutamate, serotonin, and opioids, which contribute to the balance of the central nervous system (CNS)^4^. Acupuncture facilitated synaptic dopamine release in a mouse model of 1-methyl-4-phenyl-1,2,3,6-tetrahydropyridine-induced Parkinson’s disease^5^. Acupuncture, including manual acupuncture, electroacupuncture (EA), transcutaneous electrical nerve stimulation, and laser stimulation at PC6, can reduce the incidence of postoperative nausea and vomiting, which is a similar effect to that observed from the use of antiemetics^6^. Acupuncture at PC6 modulates activity in the cardiovascular system by influencing the brain to secrete inhibitory neurotransmitters such as GABA, opioids, and 5-hydroxytryptamine to attenuate the activity of sympathetic neurons. This effect of acupuncture involves a long-loop pathway that includes the hypothalamic rostral ventrolateral medulla, arcuate nucleus, ventrolateral periaqueductal gray, and medullary raphe^7^. EA at PC6 can induce inhibition of GABA transmission to the dorsal motor nucleus of the vagus and thereby reduce the inhibition of efferent vagal motor fiber, resulting in an increase of gastric motility^8^. Therefore, acupuncture at PC6 can produce a protective cardiovascular effect and increase gastric motility related to the inhibition of GABA. Acupuncture at ST36 during the remaining needle phase can increase motor cortical excitation and reduce motor cortical inhibition through transcranial magnetic stimulation^9^. A transcriptomic analysis in mice revealed that EA at ST36 involves the GABAergic neurotransmission network of the brain^10^. Collectively, these findings indicate that points are specific and that the acupuncture effect is related to neurotransmitter transmission.

Different frequencies of EA applied to body specific point can induce the release of different neuropeptides in central nervous system, e.g. 2 Hz EA mediates μ receptor to induce the release of β-endorphin, endomorphin and enkephalins, 100 Hz EA is via κ receptor to cause the release of dynorphin, whereas 15 Hz EA is through μ, δ and κ receptors to induce the release of β-endorphin, endomorphin, enkephalins and dynorphin^11^. Our previous studies have known that 2 Hz EA applied to ST36 produces more sustained effect in reducing heart rate than 100 Hz EA in human^12^. By consecutive stimulation, EA at Dazhui (CV14) and Baihui (CV20) points 1 day after reperfusion then once daily for 6 consecutive days can reduce infarction volume and neurological deficit^13^, and also can increase long-term potentiation^14^ in ischemia–reperfusion injured rats.

Therefore, this study aimed to investigate the point specificity of 2 Hz EA at ST36 and PC6 for 5 consecutive days in Sprague–Dawley (SD) rats to evaluate the neurotransmitter changes in the cerebral cortex, hippocampus, and hypothalamus by examining their metabolomic profiles.

## Results

### Differences between the sham, PC6, and ST36 groups in the cerebral cortex, hippocampus, and hypothalamus according to PLS-DA

**Figure 1:**
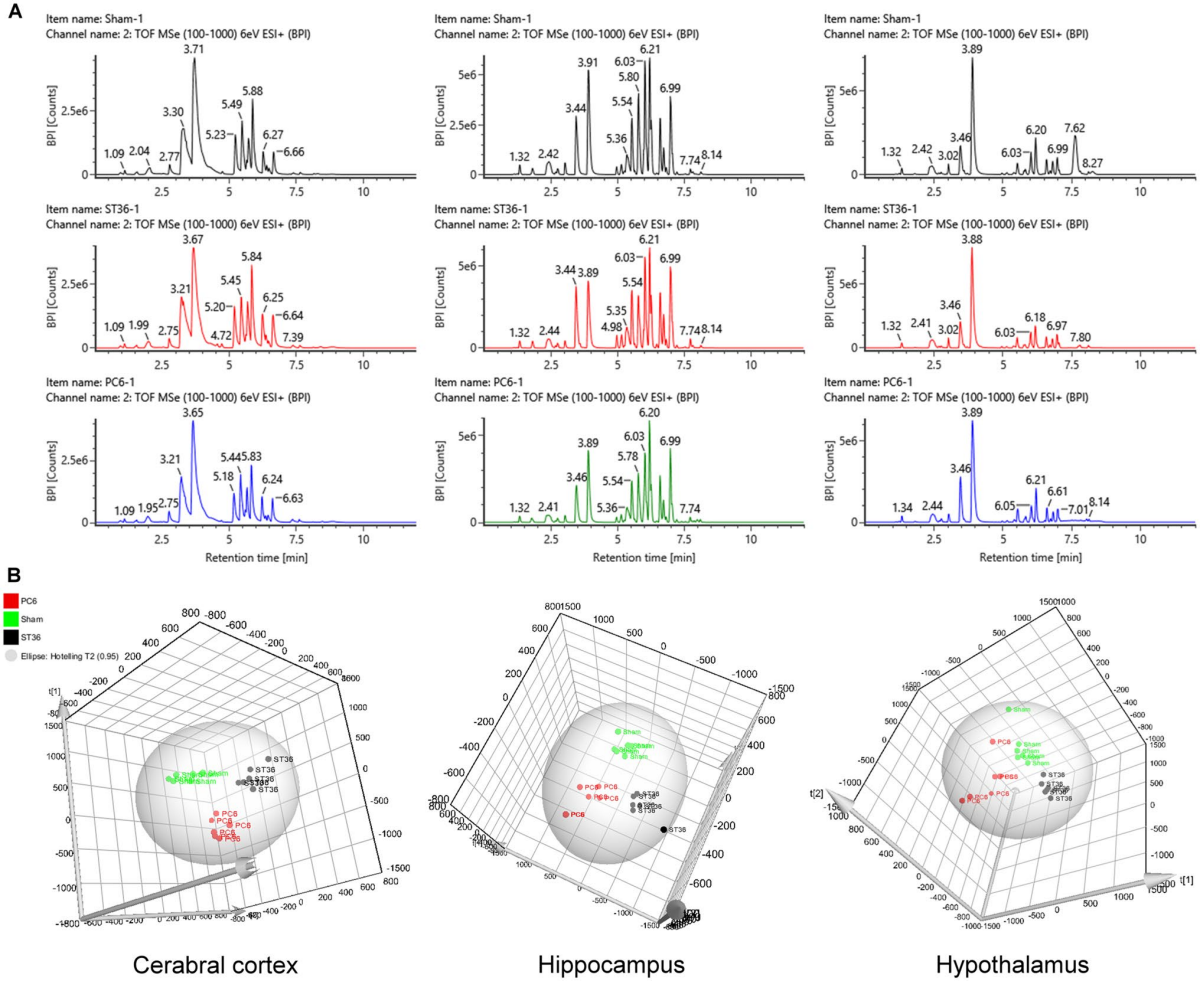
Metabolite signatures of acupoints. (**A**) Base-peak-ion chromatograms of electroacupuncture in the sham, ST36, and PC6 groups for the cerebral cortex, hippocampus, and hypothalamus, respectively. (**B**) PLS-DA plot of the cerebral cortex, hippocampus, and hypothalamus of rats presenting the difference of metabolite profiles in the sham, ST36, and PC6 groups.

Dansylated brain extracts of the cerebral cortices, hippocampus, and hypothalamus were resolved by liquid chromatography–mass spectrometry and presented as a base peak ion chromatogram (Fig. 1A). All data were further subjected to Progenesis QI for PLS-DA analysis. The distribution of each sample in the PLS-DA plot indicated differences of metabolite contents among the sham, PC6, and ST36 groups in the cerebral cortices, hippocampus, and hypothalamus (Fig. 1B).

### Effect of EA at ST36 and PC6 on neurotransmitters in the cerebral cortex

Multiple small molecular neurotransmitters were further examined by measuring the integrated chromatogram peak area in the cerebral cortex (Table 1).

**Table 1:** Summary of acquired signal counts from the integrated chromatogram peak area in the cerebral cortex.

Peak area (signal counts)	Adenosine	Adrenaline	Arginine	GABA	Glutamate	Glycine
Sham	8,506	12,562	1,825,642	36,942,871	33,059,440	21,626,500
ST36	5,192	16,702	1,723,435	34,450,560	29,231,457	21,324,986
PC6	3,640	10,330	1,487,886	26,374,864	26,862,444	15,176,943
Sham RSD (%)	23.3	3.0	16.8	17.1	22.3	13.4
ST36 RSD (%)	17.9	19.1	16.8	8.7	10.4	10.7
PC6 RSD (%)	23.7	12.3	22.7	18.4	15.4	16.9
ST36 versus Sham *p* =	0.00897	0.0178	0.54215	0.31951	0.18274	0.8175
PC6 versus Sham *p* =	0.0058	0.00734	0.15344	0.04766	0.2137	0.02532
PC6 versus ST36 *p* =	0.07712	0.01816	0.67197	0.07508	0.54096	0.05417

**Figure 2:**
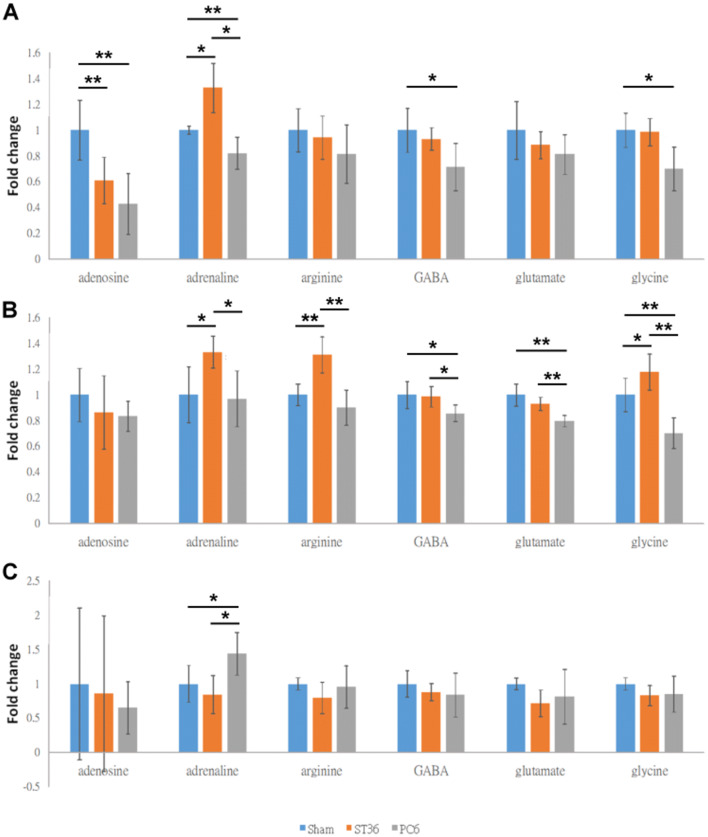
Neurotransmitter changes induced by electroacupuncture at ST36 and PC6. The relative levels of six molecules in the (**A**) cerebral cortex, (**B**) hippocampus and (**C**) hypothalamus were determined by LC–MS analysis. GABA, γ-aminobutyric acid; **p < 0.05; ***p < 0.01.

The adenosine levels of the cerebral cortices were greater in the sham group than in the ST36 and PC6 groups (both *p* < 0.01; Fig. 2A), whereas the adenosine levels of the cerebral cortices were similar between the ST36 and PC6 groups (*p* > 0.05; Fig. 2A). The adrenaline levels in the cerebral cortices were greater in the ST36 group than in the sham and PC6 groups (both *p* < 0.05; Fig. 2A). The adrenaline levels of the cerebral cortices were lower in the PC6 groups than sham (*p* < 0.01; Fig. 2A). The arginine levels of the cerebral cortices were not significantly different among the sham, ST36, and PC6 groups (all *p* > 0.05; Fig. 2A). The GABA levels of the cerebral cortices were greater in the sham and ST36 groups than in the PC6 groups (both *p* < 0.05; Fig. 2A), whereas the GABA levels of the cerebral cortices were similar between the sham and ST36 groups (*p* > 0.05; Fig. 2A). The glycine levels of the cerebral cortices were greater in the sham (*p* < 0.05) and ST36 groups than in the PC6 groups (Fig. 2A), whereas the glycine levels of cerebral cortices were similar between the sham and ST36 groups (*p* > 0.05; Fig. 2A). The glutamate and arginine levels of the cerebral cortices were not significantly different among the three groups (all *p* > 0.05; Fig. 2A).

### Effect of EA at ST36 and PC6 on the neurotransmitters in the hippocampus

A set of signals for neurotransmitters was also obtained from the hippocampus samples during examination of the cerebral cortex (Table 2).

**Table 2:** Summary of acquired signal counts from the integrated chromatogram peak area in the hippocampus.

Peak area (signal counts)	Adenosine	Adrenaline	Arginine	GABA	Glutamate	Glycine
Sham	16,610	275,884	2,249,246	5,573,497	5,869,623	7,692,728
ST36	14,344	367,814	2,952,275	5,485,762	5,457,317	9,060,471
PC6	13,859	267,383	2,027,722	4,777,837	4,669,993	5,411,060
Sham RSD (%)	20.7	21.8	8.4	10.5	8.5	12.9
ST36 RSD (%)	28.6	12.4	13.8	8.0	5.2	14.0
PC6 RSD (%)	11.5	21.7	13.8	6.3	4.4	11.7
ST36 versus Sham *p* =	0.31763	0.01169	0.00972	0.65919	0.12033	0.01361
PC6 versus Sham *p* =	0.03762	0.62355	0.0744	0.01746	0.00063	0.00327
PC6 versus ST36 *p* =	0.80332	0.02465	0.00183	0.02681	0.00215	0.00268

The adenosine levels of the hippocampi were similar among the groups (all *p* > 0.01; Fig. 2B). The adrenaline levels of the hippocampi in the ST36 group were greater than those in the sham and PC6 groups (both *p* < 0.05; Fig. 2B), whereas the adrenaline levels were similar between the sham and PC6 groups (*p* > 0.05; Fig. 2B). The arginine levels of the hippocampi in the ST36 group were greater than those in the sham and PC6 groups (both *p* < 0.01; Fig. 2B), whereas the arginine levels were similar between the sham and PC6 groups (*p* > 0.05; Fig. 2B). The GABA levels of the hippocampi in the sham and ST36 groups were greater than those in the PC6 groups (both *p* < 0.05; Fig. 2B), whereas the GABA levels of the hippocampi were similar between the sham and ST36 groups (*p* > 0.05; Fig. 2B). The glycine levels of the hippocampi in the sham and ST36 groups were greater than those in the PC6 groups (both *p* < 0.01; Fig. 2B), and the glycine levels of the hippocampi were lower in the sham than ST36 groups (*p* < 0.05; Fig. 2B). The glutamate levels of the hippocampi in the sham and ST36 groups were greater than those in the PC6 group (both *p* < 0.01; Fig. 2B), whereas the glutamate levels of hippocampi were similar between the sham and ST36 groups (*p* > 0.05; Fig. 2B).

### Effect of EA at PC6 and ST36 on neurotransmitters in the hypothalamus

Finally, data for the same set of neurotransmitters were acquired from the hypothalamus samples (Table 3).

**Table 3:** Summary of acquired signal counts from the integrated chromatogram peak area in the hypothalamus.

Peak area (signal counts)	Adenosine	Adrenaline	Arginine	GABA	Glutamate	Glycine
Sham	6,422	28,119	516,814	798,703	157,116	1,384,941
ST36	5,509	23,656	410,340	701,097	112,830	1,147,739
PC6	4,173	40,450	493,100	668,345	127,710	1,177,702
Sham RSD (%)	110.5	26.9	9.0	18.6	8.3	8.6
ST36 RSD (%)	113.5	27.4	23.0	12.3	19.2	14.4
PC6 RSD (%)	38.0	31.1	30.7	32.0	39.8	25.9
ST36 versus Sham *p* =	0.10479	0.21463	0.07938	0.11025	0.0201	0.01504
PC6 versus Sham *p* =	0.50662	0.04063	0.73616	0.1724	0.22437	0.22313
PC6 versus ST36 *p* =	0.65114	0.00342	0.36351	0.62593	0.52444	0.80948

The adenosine, arginine, glutamate, GABA, and glycine levels of the hypothalamus were similar among the three groups (all *p* > 0.05; Fig. 2C). The adrenaline levels of the hypothalamus were greater in the PC6 group than in the sham and ST36 groups (*p* > 0.05), whereas the adrenaline levels were similar between the sham and PC6 groups and between the sham and ST36 groups (both *p* > 0.05; Fig. 2C).

## Discussion

**Figure 3:**
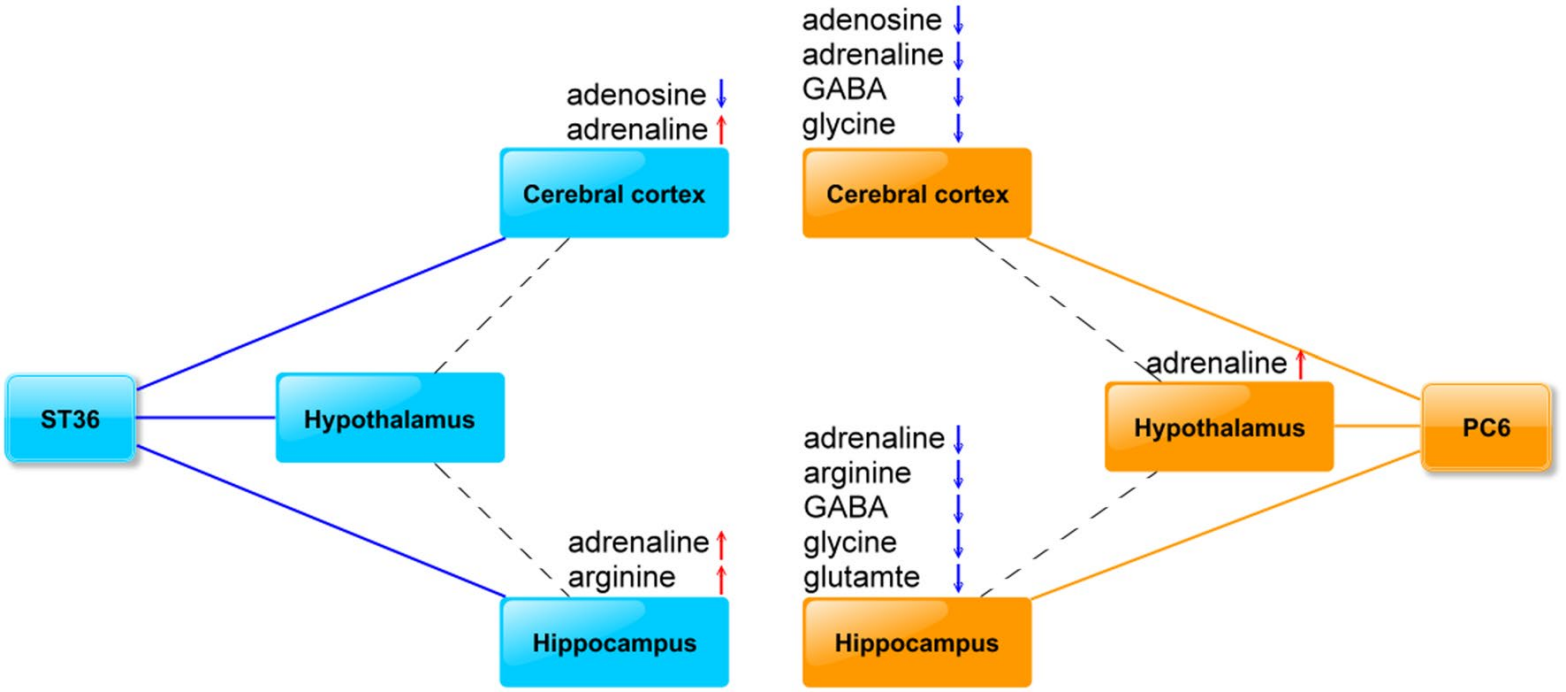
The summary of differential effects on the putative meridian paths between ST36 and PC6. The linkage of dash line implies the potential role of hypothalamus in coordinating modulation of metabolic change in cerebral cortex or hippocampus by referring the previous studies^31,32^.

The results of PLS-DA in this study indicated metabolite differences among the sham, ST36, and PC6 groups in the cerebral cortices, hippocampi, and hypothalamus. This finding suggested that the signals from the sham, ST36, and PC6 points were transmitted through different pathways according to their specific location in the cerebral cortex, hippocampus, and hypothalamus (Fig. 3). In addition, our results also indicated that EA at ST36 increased adrenaline levels in the cerebral cortex and hippocampus, whereas EA at PC6 could not produce similar results. EA at PC6 could reduce the GABA and glycine levels of the cerebral cortex and hippocampus, whereas EA at ST36 could not produce similar results, suggesting that the EA at ST36 and PC6 could cause different changes in the neurotransmitters. Collectively, the results were consistent with the idea that different points modulate the activity of a corresponding brain area and have specificity^1^. GABA is a neurotransmitter that can rapidly inhibit synaptic transmission. The GABA system has been widely used as a target in the CNS for various drugs, such as anxiolytic, sedative–hypnotic, and anticonvulsant medications^15,16^. Glycine is a secondary rapid inhibitory neurotransmitter, and its receptor involves motor reflexes and nociceptive pathways, especially in the spinal cord^15,16^. GABA-A controls inhibitory synaptic neurotransmission, whereas glutamate controls excitatory synaptic neurotransmission. A reduction in the number of GABA-A receptors reduces synaptic inhibition, and an increase in the number of glutamatergic receptors causes an imbalance between excitation and inhibition, resulting in epileptic seizure^17^. GABA dysfunction and glycine disruption involve the mechanism of neuropathic pain^18^. The results of the present study indicated that EA at PC6 reduces GABA and glycine levels in the cerebral cortex and hippocampus (Fig. 2A, B). Further studies are required to determine whether the reduction of GABA and glycine by EA at PC6 results in epileptic seizure or pain thresholds to cause seizures or neuropathic pain to occur.

Additionally, our present results also indicated that the glutamate levels in the hippocampus were also lower in the PC6 group than in the sham and ST36 groups (Fig. 2B). Glutamate is mainly an excitatory neurotransmitter in the mammalian CNS. Glutamatergic excitotoxicity plays a critical role in the pathogenesis following an ischemic stroke; additionally, it contributes to neuropsychiatric disorders such as schizophrenia and major depressive disorder^19^. The results imply that applying EA at PC6 may be feasible to treat ischemic stroke or neuropsychiatric diseases.

Our results also indicated that adenosine levels of the cerebral cortices in the ST36 and PC6 groups were lower than those in the sham group (Fig. 2A). Adenosine is a neurotransmitter that serves as an endogenous distress signal and plays a modulatory role in tissue damage and repair in the nervous system^20,21^. The formation of adenosine mainly results from the breakdown of intracellular or extracellular adenine nucleotides when cell-membrane damage occurs, such as when transient global ischemia causes a massive increase in the extracellular adenosine triphosphate (ATP), which results in the rapid formation of extracellular adenosine. By contrast, extracellular adenosine levels decrease with the increase in intracellular adenosine formation^21,22^. Acupuncture may induce increases in extracellular ATP, adenosine diphosphate, adenosine monophosphate, and adenosine levels; however, the increase in adenosine level only lasts for a short duration because of the facilitated uptake of nucleoside transporters^21,23^. Collectively, these findings indicate that the reduction of adenosine levels in the ST36 and PC6 groups may be attributable to the movement of adenosine from the extracellular to intracellular space.

Our results also indicted that the adrenaline levels of the cerebral cortices and hippocampi in the ST36 group were greater than those in the sham and PC6 groups (Fig. 2A, B). Adrenaline is one of the principal neurotransmitters in the CNS and is involved in many CNS functions. It is suggested that noradrenaline can control the neuronal activity of the cerebral cortex or amygdala in the consolidation process of long-term memory formation^24^. Adrenaline can enhance hippocampal glucose metabolism after stress to support stress-related memory processing^25^. Therefore, EA at ST36 is perhaps more suitable than EA at PC6 for improving memory.

From the results, arginine levels in the hippocampus were greater in the ST36 group than in the PC6 group, whereas the arginine levels in the cerebral cortex were not significantly different between the two groups (Fig. 2A, B). l-arginine involves nitric oxide (NO) synthase, which is related to the generation of endogenous NO. l-arginine induces vasodilation, and its long-term oral administration can alleviate the clinical symptoms of cardiovascular disorders^26^. NO levels are elevated in acupoints, inducing vasodilation and leading to an increase in local blood flow and also contributes to the specificity of acupoints^27^. The change of arginine level in these results partly support to explain of specificity of acupoints.

One of our results requires further explanation, namely why no significant difference was observed in the adenosine, arginine, GABA, and glycine levels of the hypothalamus among the three groups, except for higher adrenaline levels of the hypothalamus in the PC6 group than in the ST36 group (Fig. 3). The specificity of acupoints and the meridian in the hypothalamic paraventricular nucleus was reported in a study using acupuncture stimulation at different 33 points^28^. EA at PC6 or ST36 can elevate gonadotropin-releasing hormone (GnRH) mRNA levels in the hypothalamus of female rats^29^. GnRH acts as a primary brain signal in the hypothalamic–pituitary–ovarian axis response to the release of follicle-stimulating hormone, luteinizing hormone, and neurotransmitters and neuropeptides such as GABA and glutamate^29,30^. The functional connectivity between the amygdala and hypothalamus is observed in patients with carpal tunnel syndrome subjected to acupuncture stimuli^31^. The hypothalamus centrally communicates acupuncture stimulation because the 2 Hz EA at LI4 can induce hypothalamus activation, which in turn extends to the insula, anterior cingulate cortex, and other brain regions^32^. Although acupuncture stimulation reaches hypothalamus, only moderate change has been observed on these neurotransmitters in this study. Therefore, hypothalamus may mainly coordinate signals and transmit to the cerebral cortex and hippocampus. Considering all of these findings together, we suggest that an acupuncture signal transfers from the point to the hypothalamus, after which it transfers to the cerebral cortex and hippocampus, producing physiological action possibly playing a partially critical role in acupuncture stimulation, but further study is still needed..

In conclusion, our results indicated differences among the sham, PC6, and ST36 groups in the cerebral cortex, hippocampus, and hypothalamus. EA produces different changes at ST36 and PC6 in the aforementioned neurotransmitters in the cerebral cortex and hippocampus, suggesting point specificity. The hypothalamus is a characterized central expression of acupuncture stimulation; some signals from acupuncture stimulation extend to other brain areas, possibly through the hypothalamus, but further study is needed in the future.

## Methods

### Animals

Male SD rats, weighing 200–300 g, were purchased from BioLASCO Taiwan Co., Ltd. The rats were kept at the animal center of China Medical University (CMU) in a 12:12 h light–dark cycle environment. The room temperature was controlled at 25 ± 1 °C. The rats had free access to food and sufficient water. The laboratory use of animals was approved by the Institutional Animal Care and Use Committee of CMU, and the Guide for the Use of Laboratory Animals (National Academy Press) was followed.

### Grouping

A total of 18 SD rats were randomly divided into 3 groups, with 6 rats in each group. Rats in the sham group were anesthetized with 2% isoflurane, and acupuncture needles were subcutaneously inserted into their bilateral Zusanli (ST36) (cathode) and Shangjuxu (ST37) (anode), which have similar characteristics to those in humans. Thereafter, the needles were connected to a Trio 300 electrical stimulator (Grand Medical Instrument Co. Ltd, Ito, Japan), but electrical stimulation was not administered. This routine was performed 20 min each day for 5 consecutive days; finally, the rats were sacrificed immediately after final EA under 3–5% isoflurane anesthesia, and the rat brains were collected for metabolomic analyses. Rats in the Zusanli (ST36) group underwent the same procedure as those in the sham group, except the acupuncture needles were inserted intramuscularly at ST36 (cathode) and ST37 (anode) and an electric stimulation of 2 Hz (constant rectangle in output mode and 150 μs in wave width) was administered. The intensity of electrical stimulation was sufficient to cause visible muscle twitching. Rats in the Neiguan (PC6) group underwent the same procedure as those in the Zusanli group, except the acupuncture needles were inserted in the bilateral PC6 (cathode) and PC5 (anode).

### Metabolite sample preparation and derivatization

Rat tissue samples were processed with similar procedures as our previous work^33^. Cerebral cortex, hippocampus, and hypothalamus tissues were respectively homogenized using 1.0-mm zirconium oxide bead grinding with a ratio of 1 mg of tissue to 10 μL of ultrapure water and then centrifuged at 14,000 rpm for 10 min. Each supernatant was collected as an aliquot of 100 μL and mixed with 300 μL of 100% methanol. After 14,000 rpm centrifugation for 10 min, 150 μL of supernatant from methanol extraction was transferred to a new microtube and then subjected to vacuum drying. Each dry metabolite sample was dissolved in a reaction solution consisting of 16 μL of ultrapure water, 2 μL of 0.5 M carbonate buffer (pH 9.4), and 2 μL of 10 mg/mL dansyl chloride prepared in acetone. The reaction was conducted at 60 °C for 2 h, and then 80 μL of ultrapure water was added before the solution was incubated for 30 min at 60 °C. After being subjected to 14,000 rpm centrifugation for 10 min, the supernatant of the dansylated sample was transferred to an insert vial and kept in an autosampler at 10 °C for further analysis.

### Liquid chromatography–mass spectrometry

Samples were analyzed by our previously established methods^33^. The liquid chromatography–mass spectrometry (LC–MS) system consisted of an ultraperformance liquid chromatography (UPLC) system (ACQUITY UPLC I-Class, Waters) and an ESI/APCI source of 4 kDa quadrupole time-of-flight mass spectrometer (Waters VION, Waters). A BEH C18 column (2.1 × 100 mm, Walters) was used to perform reversed-phase LC. The flow rate was set at 0.2 mL/min with a column temperature of 35 °C and 5-μL sample injection. The sample contents were eluted by 99% mobile phase A (ultrapure water + 0.1% formic acid) and 99% mobile phase B (100% methanol + 0.1% formic acid), then held at 1% B for 0.5 min, raised to 90% B at 5.5 min, held at 90% B for 1 min, and then lowered to 1% B at 1 min. The column was equilibrated by pumping 1% B for 4 min. An LC–MS chromatogram was acquired using ESI + mode under the following conditions: capillary voltage of 2.5 kV, source temperature of 100 °C, desolvation temperature of 250 °C, cone gas maintained at 10 L/h, desolvation gas maintained at 600 L/h, and acquisition by MS^E^ mode with a range of 100–1,000 *m*/*z* and a 0.5-s scan time. The acquired data were processed using UNIFI software (Waters) to summarize the integrated area of signals, and Progenesis QI with EZinfo (Waters) was used to perform partial least squares discriminant analysis (PLS-DA).

### Statistical analysis

All data are expressed as the mean ± standard error. Significant differences were analyzed using one-way analysis of variance followed by Tukey’s test (post hoc). A* p* value less than 0.05 was considered statistically significant.

### Ethics statement

The protocol was approved by the Animal Care and Use Committee of China Medical University (study on the relationship among acupoints, the brain, and visceral organs; CMUIACUC-2017-349).

### Consent for publication

This study did not include any human data.
